# Diabetic Women Suffer More Years of Life Lost Than Diabetic Men

**DOI:** 10.1155/2014/208369

**Published:** 2014-12-03

**Authors:** Zhi-Jiang Zhang, Genming Zhao, Chuanhua Yu, Yongyi Bi, Qingjun Zhang, Yiqing Song

**Affiliations:** ^1^School of Public Health, Wuhan University, 185 Donghu Road, Wuhan 430071, China; ^2^School of Public Health, Fudan University, Shanghai 200032, China; ^3^Hubei Center for Disease Prevention and Control, Wuhan 430079, China; ^4^Department of Epidemiology, Indiana University, Richard M. Fairbanks School of Public Health, IN 46202, USA

We read with interest the recent paper by Harjutsalo and colleagues [1] which reported that the standardized mortality ratio due to ischemic heart disease was higher in women than in men in the cohort of type 1 diabetes: within the early-onset (0–14 years) cohort, the standardized mortality ratio was 52.8 (95% confidence interval 36.3–74.5) in women compared with 12.1 (9.2–15.8) in men; within the late-onset (15–29 years) cohort, the standardized mortality ratio was 15.8 (11.8–20.7) in women compared with 5.0 (4.3–5.8) in men.

Their results are in agreement with epidemiologic data from China. Based on the death registry data from Shanghai CDC, female diabetic patients (both type 1 and type 2) suffer more Years of Life Lost (YLLs) than male diabetic patients [2]. Specifically, the average of YLLs per diabetic patients, defined as the sum of YLLs divided by total number of female or male diabetic patients, was higher in female diabetic patients than male diabetic patients from 1976 to 1996 (Figure 1(a)); the average of YLLs per resident, defined as the sum of YLL divided by total number of female or male residents in Shanghai, was higher in female residents than male residents (Figure 1(b)).

An earlier population-based study in China showed that female diabetic patients were more likely to have chronic diabetic complications [3]. Of the 255 diabetic patients who were identified by fasting-glucose screening in a cross-sectional survey of 1960 adult residents enrolled through a multistage sampling scheme from 8 communities in Shanghai, the prevalence of diabetic complications (including any of diabetic retinopathy, diabetic nephropathy, diabetic foot ulcer, or diabetic cardiovascular complications) in diabetic women was higher than that in diabetic men (37.7% versus 24.8%, odds ratio 1.74, 95% confidence interval 1.01–3.06, *P* = 0.05) [3]. These two studies are, to our knowledge, the first to report the gender difference in diabetic mortality [2] and chronic diabetic complications [3] in Chinese population, supported by data from other countries [1, 4–6]. These findings suggest the loss of female gender as a protective factor in terms of life expectancy and a variety of chronic diseases, for example, cardiovascular disease, in the setting of diabetes.

In summary, diabetic women are at higher risk of chronic complication and mortality than diabetic men. Further studies investigating the underlying mechanisms are warranted More efforts are needed for controlling diabetic complications for women, for example, health education for both patients and health professionals.

## Figures and Tables

**Figure 1:**
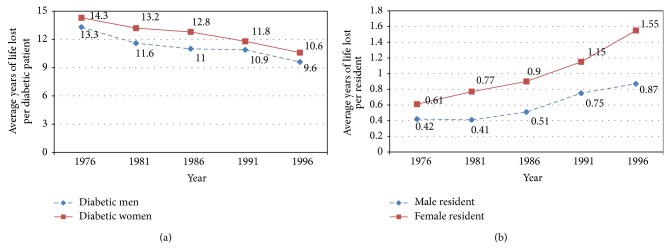
The average of Years of Life Loss (a) per diabetic patient and (b) per resident in Shanghai between 1976 and 1996.
